# Utility of Adrenal Vein Sampling to Guide Surgical Management of Hypercortisolism

**DOI:** 10.1155/crie/2720854

**Published:** 2024-12-19

**Authors:** Brandon Tran, James Y. Lim, Brian Park

**Affiliations:** ^1^Virginia Commonwealth University School of Medicine, Richmond, Virginia, USA; ^2^Department of Surgical Oncology, Oregon Health and Science University, Portland, Oregon, USA; ^3^Department of Interventional Radiology, Oregon Health and Science University, Portland, Oregon, USA

## Abstract

We report a case of successfully lateralized adrenal cortisol hypersecretion by adrenal venous sampling (AVS) and improved by surgery. AVS is a commonly used tool to guide surgical management of primary hyperaldosteronism. It can determine lateralization, leading to unilateral adrenalectomies of the correct side, or nonlateralization, which precludes surgery. The use of AVS in determining lateralization in hypercortisolism is a growing field of discussion. Currently, there is no defined or unanimous protocol behind procedural details and interpretation of results. In this report, we describe the AVS protocol at our institution for hypercortisolism, interpretation of the results, and corresponding surgical outcomes for a case of mild autonomous cortisol secretion.

## 1. Introduction

The evaluation of a patient with autonomous cortisol secretion involves localization of the source of secretion, which can frequently be asymmetric [1]. In the case of adrenal hypercortisolism, an abdominal CT can be performed to assess for adrenal adenomas or asymmetric adrenal hypertrophy, indicating a potential surgical target. Although adrenal venous sampling (AVS) is a standard procedure to determine laterality in the setting of primary hyperaldosteronism to guide surgical treatment, AVS in the setting of hypercortisolism is less clear with only a small number of case series showing its potential utility [1–3]. Nevertheless, any additional information on patients with bilateral adrenal adenomas can be beneficial to provide a more informed surgical treatment plan.

## 2. Case Presentation

**Figure 1:**
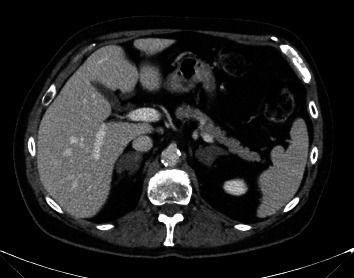
Preoperative CT scan was interpreted as benign bilateral adrenal adenomas measuring up to 3.9 cm on the right and 3.5 cm on the left, based on unenhanced attenuation <0 Hounsfield units and relative washout >40% on both sides.

**Figure 2:**
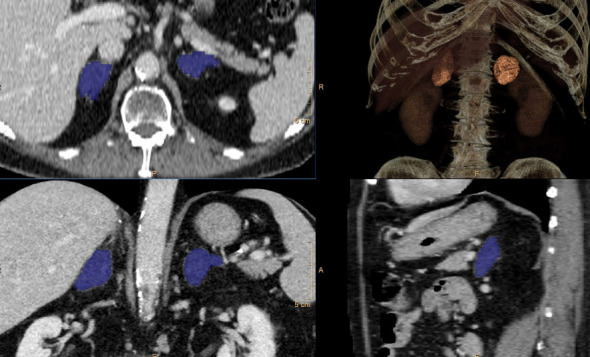
Bilateral adrenal glands were manually segmented using Philips Advance Visualization Workspace software. Right adrenal volume was 24.3 cc. Left adrenal volume was 18.4 cc. The right adrenal volume is larger than the left by 5.9 cc (24.2%).

A 74-year-old male presents with a history of hypertension, hyperlipidemia, and type 2 diabetes and was incidentally found to have bilateral adrenal adenomas while undergoing work-up for hematuria (Figure 1). He denied a history of abnormal blood pressure elevations, palpitations, headaches, diaphoresis, sudden weight gain, muscle weakness, mood instability, or easy bruising. Labs were drawn to rule out evidence of adrenal hormone excess, and his low-dose dexamethasone suppression test returned abnormal. The most recent HbA1c was 10.1%, and diabetes was controlled on metformin and sitagliptin. Further investigation revealed an elevated 24-h urine cortisol and a suppressed ACTH at 7.0 pg/mL. DEXA scan showed osteopenia of the femoral neck with *T*-score of −1.5. These lab results in combination with the CT imaging suggested mild autonomous cortisol secretion due to a functional adenoma. There was no significant asymmetry in adrenal size on imaging with 3D segmentation revealing less than 30% difference in total adrenal volume (Figure 2).

**Figure 3:**
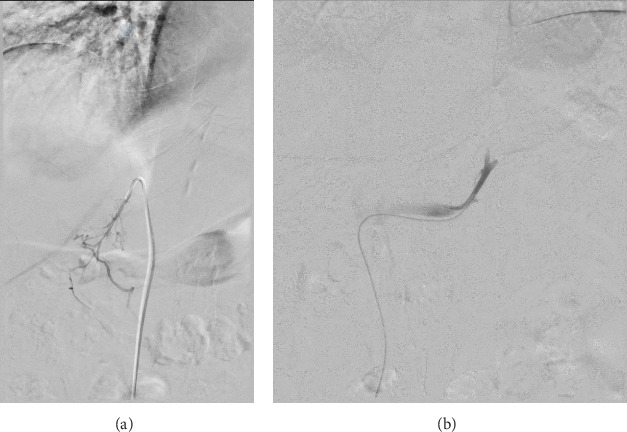
(A) Digital subtraction angiography image of a 5 French RDC catheter in right adrenal vein. Emissary veins can be seen traversing inferiorly providing angiographic confirmation of right adrenal vein catheterization. (B) Digital subtraction angiography image of 5 French SIM2 catheter in the left adrenophrenic trunk.

AVS was performed with the goal of identifying laterality and avoiding bilateral adrenalectomy. The patient was instructed to take low-dose dexamethasone, 0.5 mg every 6 h, for 24 h prior to the procedure. The procedure was performed with the patient under moderate sedation. Access was obtained via the right common femoral vein. Samples were obtained sequentially from the right and left adrenal veins (Figure 3) and infrarenal IVC. All samples were acquired within 5 min of each other.

These samples were sent for cortisol, aldosterone, epinephrine, norepinephrine, metanephrine, normetanephrine, and DHEA-S. The samples were adequate based on the cutoff ratio of the following hormones from each adrenal vein and the peripheral vein (PV) or infrarenal IVC: cortisol ratio ≥3–5, aldosterone ratio >2, metanephrine ratio >12, and epinephrine difference >100 pg/mL (1, 2).

Lateralization indices were calculated as follows:

  $$\begin{array}{c} \text{Lateralization index}=\frac{\left(\frac{\text{Cortisol}}{\text{reference hormone}}\right)\text{higher side}}{\left(\frac{\text{Cortisol}}{\text{reference hormone}}\right)\text{lower side}}. \end{array}$$

Lateralization index ≥2 was considered significant [1]. The lateralization indices suggested lateralization to the right adrenal gland (Table 1), based on aldosterone and DHEA-S as reference hormones. Additionally, there was lateralization based on adrenal-to-peripheral cortisol ratio >6.5 (28.3/1.9 = 14.89) as well as side-to-side adrenal cortisol ratio >2.3 (28.3/7.4 = 3.82), which also suggested the right adrenal as the culprit [4, 5].

The patient elected to proceed with surgery and subsequently underwent right adrenalectomy without incident. Pathology revealed diffuse adrenal cortical hyperplasia without discrete mass lesion (43 g). The AM cortisol after the surgery was normal at 17.3 µg/dL. Postoperatively, the patient had improvement in his glucose control and no subsequent symptoms of hyper- or hypocortisolism. Two months after surgery, biochemical response was appropriate with morning ACTH no longer suppressed at 62.6 pg/mL.

## 3. Discussion

Although there have been no robust studies documenting the use of AVS in guiding management decisions of Cushing's syndrome with bilateral adrenal adenomas, there have been small case series showing the benefits of AVS in decision-making [1, 3–6]. AVS has previously been established as playing a critical role in the management of primary aldosteronism [2, 7]. In this case, AVS was utilized to verify removal of the more functionally active gland. Removal of the more dominant gland, or major producer of cortisol, may allow for more immediate reduction in cardiometabolic risk and easier long-term clinical management.

Pathology revealed diffuse hyperplasia without a discrete mass lesion, representing a discrepancy from the preoperative CT report. However, hyperplasia cannot be reliably differentiated from adenoma based on imaging [8]. Though the volume of mass can suggest cortisol production as demonstrated by a study by Rubinstein et al. [3], AVS can still be beneficial in determining laterality especially when there are bilateral adrenal masses of similar volumes. Despite this, the utility of AVS in the setting of hypercortisolism and bilateral adrenal hyperplasia remains unclear. The vast majority of patients with Cushing's syndrome or mild autonomous cortisol secretion have bilateral adrenal disease. Optimal management of these patients, including surgery versus conservative management, is currently performed on an individualized approach based on patient preference, cardiometabolic risk factors, and other comorbidities.

Despite the effectiveness of AVS in this case, questions remain about the standard practice of AVS for hypercortisolism. Protocols, reference hormones, selectivity indices, and lateralization indices vary based on technical ability and institutional preferences. Some studies suggest confirming lateralization solely with a cortisol lateralization ratio >2.3 while others suggest utilizing a cortisol AV:PV gradient of >6.5 [4, 5, 9]. However, cortisol levels may vary significantly throughout the day and during periods of stress, such as undergoing a medical procedure. Preprocedural dexamethasone may mitigate these effects but dosing regimens for suppression are also varied. Additionally, comparisons of absolute cortisol values do not account for dilution of the left adrenal vein sample from influx from the left inferior phrenic vein.

Other institutions may use multiple reference hormones interchangeably or in combination with each other to confirm catheter placement and adjust for dilution from blood efflux [3]. However, the need for more reference hormones requires larger volumes of sampling, which can increase procedure difficulty as the catheters are frequently positioned in tenuous locations. There is also no standard practice for simultaneous versus sequential methods of AVS [9]. Furthermore, selectivity and lateralization indices play a significant role in determining if samples are adequate and if secretion is truly unilateral, both of which have no consensus on standard criteria or thresholds [10].

In this case, lateralization indices suggested lateralization to the right adrenal gland based on aldosterone and DHEA-S as reference hormones. It is unclear why these reference hormones demonstrated lateralization compared to the other reference hormones, and further research may elucidate which reference hormones may have the highest utility and yield in the setting of hypercortisolism. DHEA-S may not be a good reference hormone due to its long half-life of 10 h [4]. Rubinstein et al. [3] also questioned the utility of DHEA-S as a reference hormone due to its persistently low measured values across cases. There is no current standard of care for the management of mild autonomous cortisol secretion or establishment of the singular, best reference hormone.

Nevertheless, we recommend sending a variety of reference hormones at the time of sampling in case the absolute cortisol values do not satisfy adrenal-to-peripheral cortisol ratio >6.5 or side-to-side adrenal cortisol ratio >2.3. In our case, the combination of the side-to-side adrenal cortisol ratio, cortisol-to-aldosterone ratio, and cortisol-to-DHEA-S ratio suggested laterality and justified our decision to pursue unilateral adrenalectomy. Even if measured cortisol levels satisfy these thresholds, the added information from lateralization indices based on reference hormones can be beneficial for confirmation and the establishment of future standards.

## 4. Conclusion

This case illustrates how AVS may help in successfully lateralizing adrenal cortisol hypersecretion, allowing for more informed surgical decision-making. However, more studies are needed to understand how AVS fits into the management of hypercortisolism and determine standard parameters involved with AVS.

## Figures and Tables

**Table 1:** Biochemistry results and analysis of AVS.

Hormones	Right adrenal	Left adrenal	IVC	SI right	SI left	LI R/L ratio	LI L/R ratio
Cortisol (μg/dL)	28.3	7.4	1.9	14.89	3.89	**3.82**	—
Aldosterone (ng/dL)	25.6	15.6	6.7	3.82	2.33	**2.33**	—
Epinephrine (pg/mL)	2226	525	10	—	—	—	1.11
Norepinephrine (pg/dL)	1752	640	322	—	—	1.40	—
Metanephrines (nmol/L)	23.9	7.28	0.10	239	72.8	1.16	—
Nometanephrines (nmol/L)	9.2	3.11	0.46	—	—	1.29	—
DHEA-S (µg/dL)	62	53	51	—	—	**3.27**	—

*Note:* R/L and L/R ratios are to compare magnitudes of adrenal products to aid in determination of laterality. The bold values are used to highlight the reference hormones that demonstrated significant laterality that ultimately led to the decision to proceed with the right adrenalectomy.

Abbreviations: AVS, adrenal venous sampling; LI, laterality index; SI, selectivity index.

## Data Availability

Data are available upon request from the corresponding author.
